# Public awareness and attitudes toward biobank and sample donation: A regional Chinese survey

**DOI:** 10.3389/fpubh.2022.1025775

**Published:** 2022-11-23

**Authors:** Zhaolin Gao, Yanxia Huang, Fei Yao, Ziyu Zhou

**Affiliations:** ^1^Department of Anatomy and Neurobiology, School of Basic Medical Science, Central South University, Changsha, China; ^2^Hunan Key Laboratory of Ophthalmology, Central South University, Changsha, China; ^3^Center for Experimental Medicine, The Third Xiangya Hospital, Central South University, Changsha, China; ^4^The Cancer Hospital of the University of Chinese Academy of Sciences (Zhejiang Cancer Hospital), Hangzhou, China; ^5^Institute of Basic Medicine and Cancer (IBMC), Chinese Academy of Sciences, Hangzhou, China

**Keywords:** biobank, sample donation, publicity, attitude, motivation, concern

## Abstract

**Background:**

The biobank is an extraordinary aid to research and scientific progress. Public involvement in biobanks, necessary for their development, is limited due to inadequate knowledge of biobanking and concerns about sample donation. This study explores the effectiveness of different publicity methods in improving participants' willingness to donate, and assesses public motivations and concerns. It aims to identify an efficient method of improving participants' awareness of biobanking and promoting sample donation.

**Methods:**

A structured 20-item questionnaire was formulated to evaluate participants' knowledge of and attitudes toward biobanks and sample donation. In total, 1,500 questionnaires were disseminated to three groups of 500 participants who received, respectively, picture-based promotional material, text-based promotional material, or who attended a biobank-related lecture. Of these, 945 completed questionnaires were received. All the participants completed the questionnaires twice, before and after the corresponding publicity education.

**Results:**

After each of the three methods of publicity based on text, pictures and a lecture, respondents' willingness to donate samples was significantly increased (*P* < 0.001), the lecture being more effective than the other two methods (*P* = 0.001). Participants with a medical background were more willing to donate biospecimens after publicity than those without medical backgrounds (*P* < 0.005) but had common motivations for donation including altruism and aiding medical research. The main concern hindering respondents' willingness to donate was the security of personal information.

**Conclusion:**

Different types of biobank-related publicity based on text material, pictorial material and a lecture all improved respondents' willingness to donate and reduced concerns regarding sample donation. Medical background was a critical factor affecting attitudes toward sample donation after publicity. The results of this study suggest strategies that may popularize biobanks and enhance sample donation, further promoting the development of biobanks.

## Introduction

Biosamples are vital resources for modern medical and biological research which are mainly gathered *via* donors and are stored in biobanks (1). A well-established biobank offers qualified biosamples for epidemiological, clinical and pharmaceutical research (2–4). Often, the modern biobank requires pathological biosample as well as healthy biosamples, including biofluid, stool, tissues, organs or processed biosamples (5).

Recent years have witnessed an acceleration in the construction of modern biobanks due to the rapid growth of medical research (6). Biobanks are important resources for research and scientific progress which can help to uncover the more complex mechanisms of biodiversity and the physiological and pathological mechanisms that underlie the state of human health (7, 8). Since biobanks entail the collection and storage of tissue and/or blood samples as well as additional personal data, public involvement is of great importance to their progress (9, 10), but various factors limit public participation. For example, the level of awareness of biobank and sample donation has not kept pace with biobank construction (11–13). In a study on public perceptions of biobanks in Europe more than two thirds of all Europeans reported no awareness of biobanks, and only 17% were actively engaged in or had searched for information about biobanks in the past (14). Similarly, a survey on the attitude of Kaiser Permanente Colorado (KPCO) members toward biobanks, found that up to 67% of Americans had not heard of biobanks (15). A low level of awareness regarding the concept of biospecimens was also observed in American local communities. Some participants were familiar with sperm banks, blood banks, and umbilical cord banks, but had not heard the term “biobank” (16). In China, focus group studies have revealed very little knowledge about biobanks among participants. Insufficient knowledge of biobank and sample donation is unfavorable to engagement in biobanking (17, 18) and public trust is essential to foster public engagement and encourage donations (19). One study showed that a failure to obtain public trust partly contributed to failures in public biobanks' clinical-data-sharing initiatives (20). In addition, the use of terms such as “donation” and “donor” shapes a professional culture in which biobank participants are perceived as passive providers of tissue free from further considerations or entitlements (21), weakens motivation and discourages participation. Biobank-related education and publicity is the most promising solution proposed to eliminate the above barriers and improve public participation.

Effective methods of publicity can improve public awareness of biobanks and sample donation as well as relieving concerns, further encouraging public participation and promoting the development of biobanks. Text, pictures and lectures are common existing publicity methods used to transmit information and knowledge (22–24). Studies have shown that these publicity methods can to some extent improve participants' willingness to donate (22, 25, 26). However, the most effective method has not been identified. Therefore, in the present study the three publicity methods of text-based materials, picture-based materials and lectures were used to increase biobank-related knowledge among a target general population. A questionnaire on biobanks and sample donation as well as educational materials about biobanks were prepared. The questionnaire was distributed with text- or picture-based materials, or provided to attendees of a biobank-related lecture. Participants were required to respond to the questionnaire before and after receiving the publicity. The goal of this study was to address three primary questions related to biobanking: (i) Are the three biobank publicity methods similarly effective in raising the participants' willingness to donate? (ii) What factors might influence the effectiveness of text-, picture-, or lecture-based material in raising participants' willingness to donate? (iii) What motivations and concerns do members of the public hold about biobanking and donation? Since awareness of biobanking is limited among Chinese populations, the questions and publicity materials were at a basic level of knowledge about biobanks and sample donation. The study aims to identify an effective method of enhancing awareness about biobanking among the general population, further improving public donation willingness and prompting biobank development.

## Materials and methods

### Survey design, setting, and participants

This research used a cross-sectional method. Given our target population was young, we disseminated questionnaires to college students mainly through online channels, including e-mail and social network applications. To determine which education method has the greatest influence on donation willingness, we used several biobank promotion methods, including questionnaires with promotional material attached and an oral lecture. Promotional material attached to questionnaires came in two forms: picture-based and text-based. A total of 1,500 questionnaires were distributed to three groups receiving different types of publicity education: 500 participants received text-based promotional material and 500 received picture-based promotional material (distributed *via* a web chat group or e-mail), while 500 attended a lecture on biobanks. All questionnaires were evaluated, and a consensus was reached by three independent reviewers (27, 28).

### Sample size estimation

A pilot 20-item questionnaire was trialed among 40 young people to ensure all participants understood the study. We estimated that 60% of respondents would support the biosample donation (29) after obtaining a response rate of 63.2% in the test survey. To obtain a 95% confidence interval (CI) of ±2.5% (30–34) around 65%, nearly 1,400 participants were needed to be recruited in this survey. Thus, 1,500 people were recruited to participate.

### Questionnaire design and promotional materials preparation

A 20-item questionnaire was designed based on previous studies on healthy people (35, 36) to evaluate the efficiency of online publicity in helping biobank construction. Respondents' demographic information, including age, gender, nationality, career, educational background, marital status, family disease history, previous donation history, in addition to previous knowledge of biosample donation, willingness to donate, donation motivation, and concerns regarding biobanks, was gathered *via* the questionnaire. Chinese was the primary language of the questionnaire and publicity materials. The term “biosamples” in this survey refers to samples obtained from relatively non-invasive routes, such as blood, urine, feces and saliva, as well as discarded test biosamples and post-operative biosamples. We also included stem cells (such as the well-known human umbilical cord blood stem cells) in our publicity material to facilitate participants' awareness.

The promotional materials were based on biobank-related courses, lectures and promotional materials of other biobanks in China as well as materials on the internet. The materials were summarized and processed by the research team and had not been used in other biobanks in China (see Supplementary Text S1, Figure S1). They were created *ad hoc* and distributed to the participants together with the questionnaire.

### Group classification

A 5-point Likert scale was used to assess the primary outcomes (37). The options “I would certainly agree” and “I would agree” were grouped as “agree,” while the options “I am not sure,” “I would disagree,” and “I would certainly disagree” were grouped as “disagree.” Educational backgrounds were grouped into “Secondary school,” “University degree,” and “Postgraduate degree.” The options “I am not concerned” and “I am completely not concerned” were classified as “positive,” while the options “I am not sure,” “I am concerned,” and “I am very concerned” were classified as “negative.”

### Statistical analysis

Quantities and percentages were calculated from all data. To explore whether all the three methods increase participants' willingness to donate, McNemar's test was used to compare the donation rate of the same groups before and after publicity. A Chi-square test was used to compare data between groups, to determine the effect of the three methods in improving participants' willingness to donate, and to identify the most effective method. A Chi-square test was also used to analyze whether gender, residence, education, profession, or health condition influenced the effectiveness of text-based material, picture-based material, or a lecture in raising participants' willingness to donate. Fisher's exact test was applied to cases in which the expected frequencies were <5. All statistical analyses were conducted using SPSS software system (SPSS version 22).

## Results

### Participant characteristics

Of the 1,500 questionnaires distributed, 945 responses were received. Out of 500 people in each group, we received 259 (27.4%), 310 (32.8%), and 376 (39.8%) responses from those who were given biobank-related publicity based on text material, picture material, and a lecture, respectively. Most of the respondents (*N* = 793; 83.9%) came from urban areas, and most (*N* = 849; 89.8%) had a university degree or higher education. The majority of participants (*N* = 869; 92%) were in good health and had never visited a hospital for disease treatment. When asked whether they were familiar with biobanks and willing to donate their samples, only 20.7% of participants knew about biobanks and only 9.6% stated that they would like to donate their samples. The participants' baseline characteristics are listed in Table 1.

**Table 1:** Baseline characteristics of the participants.

**Participant characteristic**	**Population *N***	**Percentage (%)**
**Gender**		
Male	424	44.9
Female	521	55.1
**Residence**		
Urban	793	83.9
Rural	152	16.1
**Education**		
Secondary school	96	10.2
University degree	619	65.5
Postgraduate degree	230	24.3
**Profession**		
Medical	464	49.1
Non-medical	481	50.9
**Health condition**		
Healthy	869	92
Diseased ever	76	8
**Familiar with biobank and specimen donation previously**		
Yes	196	20.7
No or not sure	749	79.3
**Specimen donation**		
Willing	91	9.6
Unwilling	854	90.4

Data presented as number and frequency.

### Publicity method effectiveness

**Figure 1:**
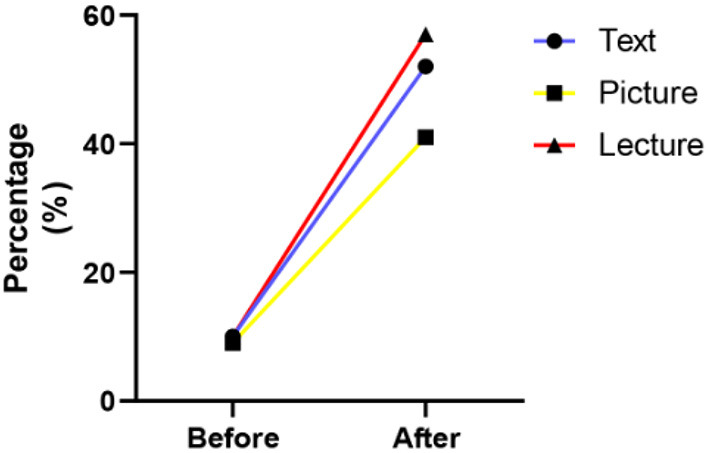
The percentage of participants willing to donate biospecimen before and after text-, picture- and lecture-based publicity.

We next explored whether the three methods of biobank publicity could enhance the participants' willingness to donate samples and which was the most effective. In the text-based material publicity group, 10% of respondents stated that they would like to donate their biospecimens before publicity, while the rate increased to 52.9% after publicity (*P* < 0.001). The picture-based material publicity also significantly increased the participants' willingness to donate (41.3 vs. 8.7%, respectively, *P* < 0.001). The lecture also prompted participants' awareness of biobanks and willingness to donate their biospecimens, donation rate increasing from 10.1 to 56.6% after attending the lecture on biobanks (*P* < 0.001). A comparison of the effectiveness of the three methods in raising willingness to donate revealed that text-based publicity and lecture-based publicity were more effective than picture-based material publicity (*P* = 0.001), with the highest increase in donation rate observed in participants who received lecture-based publicity (Figure 1). The details are shown in Table 2.

**Table 2:** The effectiveness of different publicity methods in raising people's donation willingness.

**Publicity**	**Total** ***N***	**Before**		**After**		***P^1^***	**Increased people (AP vs. BP)** ***N*** **(%) ^c^**	***P^2^***
		**WTD** ***N*** **(%)^c^**	**NWTD** ***N*** **(%)^c^**	**WTD** ***N*** **(%)^c^**	**NWTD** ***N*** **(%)^c^**			
Text	259	26 (10)	233 (90)	137 (52.9)	122 (47.1)	< 0.001	111 (42.9)	0.001
Picture	310	27 (8.7)	283 (91.3)	128 (41.3)	182 (58.7)	< 0.001	101 (32.6)	
Lecture	376	38 (10.1)	338 (89.9)	213 (56.6)	163 (43.4)	< 0.001	175 (46.5)	

WTD, willing to donate; NWTD, not willing to donate; BP, Before publicity; AP, After publicity.

c Data presented as number and frequency.

1 McNemar's test was used to compare the donation rate of the same groups before and after publicity (WTD rate after publicity vs. before publicity within groups).

2 Chi-square test was used for between-group comparisons to compare the effect of the three methods in improving participants' willingness to donate.

### Medical background as a factor in willingness to donate

We further analyze whether gender, residence, education, profession, and health condition influenced the effectiveness of text-based material, picture-based material, and lecture in raising participants' willingness to donate. All three types of publicity significantly improved the willingness of respondents, including both males and females, people living in both rural and urban areas, people with both secondary school education and university degrees and above, people both with and without medical backgrounds, as well as both healthy people and those with a history of disease. Willingness to donate increased more among participants with than without a medical background, increasing by 55.2 and 32.9% (*P* < 0.001), respectively after text-based publicity, and by 40.2 and 24.9% (*P* = 0.004), respectively after picture-based publicity, and by 56.0 and 36.7% (*P* < 0.001), respectively after lecture-based publicity. Residence was another factor influencing the effect of picture-based publicity on participants' donation willingness (*P* = 0.007). No difference was found in the impact of publicity on willingness to donate between participants of different genders, education backgrounds, or health conditions. The details are shown in Table 3.

**Table 3:** Factors influencing the effectiveness of text-based, picture-based, and lecture-based publicity in improving willingness to donate.

**Factors**	**Text-based publicity**				**Picture-based publicity**				**Lecture-based publicity**			
	**(WTD)**				**(WTD)**				**(WTD)**			
	**Total** ***N***	**BP** ***N*** **(%)*^1^***	**AP** ***N*** **(%)*^1^***	***P^2^***	**Total *N***	**BP** ***N*** **(%)*^1^***	**AP** ***N*** **(%)*^1^***	***P^2^***	**Total** ***N***	**BP** ***N*** **(%)*^1^***	**AP** ***N*** **(%)*^1^***	***P^2^***
**Gender**												
Male	116	9 (7.8)	62 (53.4)	0.407	148	15 (10.1)	61 (41.2)	0.59	160	19 (11.9)	92 (57.5)	0.759
Female	143	17 (11.9)	75 (52.4)		162	12 (7.4)	67 (41.4)		216	19 (8.8)	121 (56.0)	
**Residence**												
Urban	38	3 (7.9)	15 (39.5)	0.128	54	5 (9.3)	31 (57.4)	0.007	60	5 (8.3)	28 (46.7)	0.164
Rural	221	23 (10.4)	122 (55.2)		256	22 (8.6)	97 (37.9)		316	33 (10.4)	185 (58.5)	
**Education**												
Secondary school	27	1 (3.7)	13 (48.1)	0.86	26	4 (15.4)	16 (61.5)	0.123	43	7 (16.3)	29 (67.4)	0.519
University degree and above	232	25 (10.8)	124 (53.4)		284	23 (8.1)	112 (39.4)		333	31 (9.3)	184 (55.3)	
**Profession**												
Yes	116	10 (8.6)	74 (63.8)	< 0.001	157	17 (10.8)	80 (51.0)	0.004	191	16 (8.4)	123 (64.4)	< 0.001
No	143	16 (11.2)	63 (44.1)		153	10 (6.5)	48 (31.4)		185	22 (11.9)	90 (48.6)	
**Health condition**												
Healthy	239	23 (9.6)	122 (51.0)	0.107	290	25 (8.6)	118 (40.7)	0.464	340	37 (10.9)	195 (57.4)	0.931
Diseased ever	20	3 (15)	15 (75.0)		20	2 (10.0)	10 (50.0)		36	1 (2.8)	18 (50.0)	

WTD, willing to donate; BP, Before publicity; AP, After publicity.

1 Data presented as number and frequency.

2 Chi-square test was used to analyze whether gender, residence, education, profession, and health condition influenced the effectiveness of each publicity method in raising participants' willingness to donate. Fisher's exact test was applied for those cases in which the expected frequencies were < 5.

### Respondents' main motivations for sample donation

**Figure 2:**
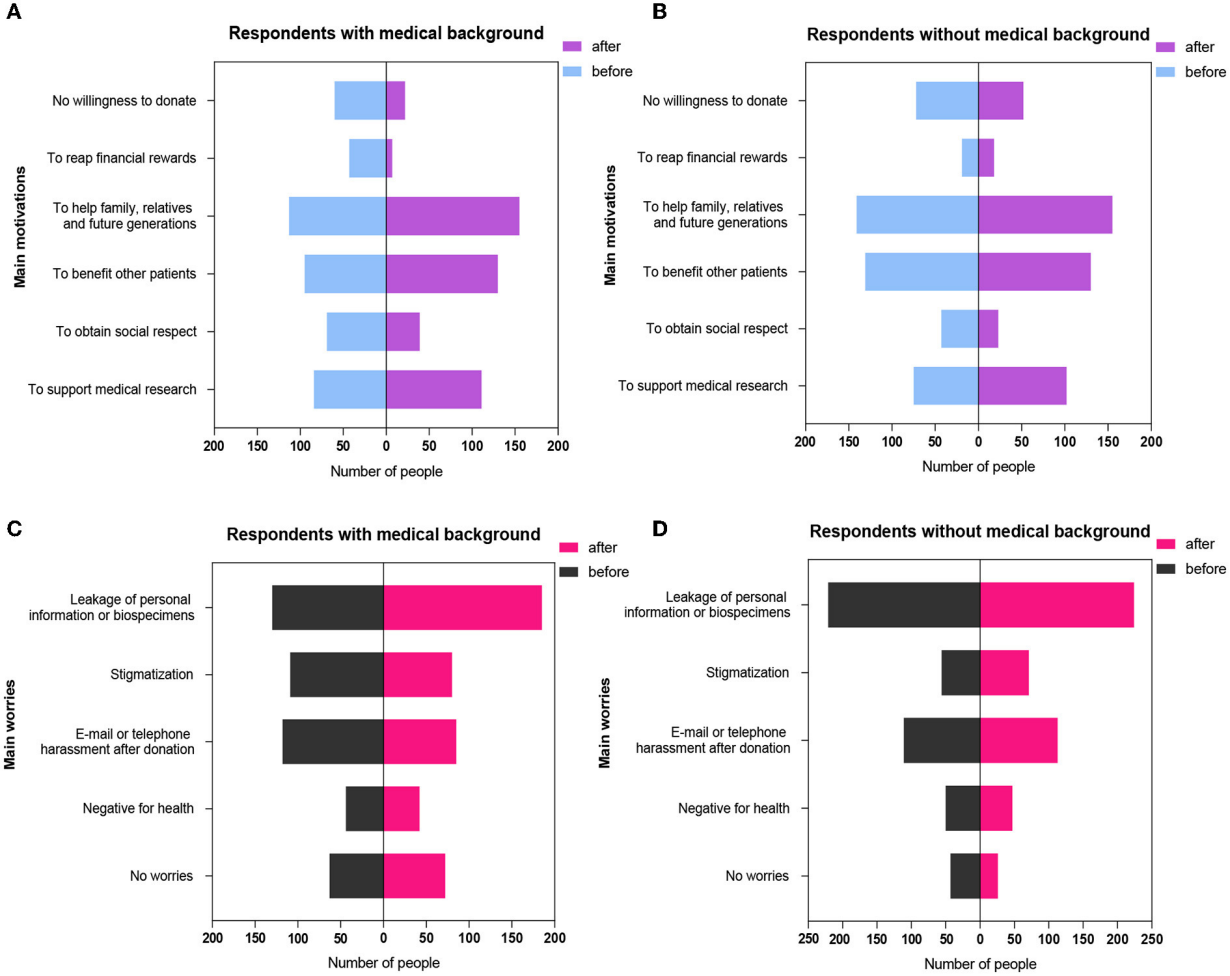
The changes in main motivations and concerns of respondents about sample donation before and after publicity. **(A)** The changes in motivations toward sample donation among respondents with medical background before and after publicity. **(B)** The changes in motivations toward sample donation among respondents without medical background before and after publicity. **(C)** The changes in concerns about sample donation among respondents with medical background before and after publicity. **(D)** The changes in concerns about sample donation among respondents without medical background before and after publicity.

Our study also investigated respondents' motivations to donate. We observed that regardless of medical backgrounds “To help family, relatives, and future generations,” “To benefit other patients,” and “To support medical research” were significant motivations for donating samples. Before participants received the publicity, those with medical backgrounds who chose the above motivations accounted for 24.4, 20.5, and 18.1%, respectively, while those without medical backgrounds who chose the above reasons accounted for 29.3, 27.2, and 15.6%, respectively. Fewer participants chose “To obtain social respect” and “To reap financial rewards.” Biobank and sample donation publicity prompted more respondents with medical backgrounds to choose “To help family, relatives and future generations,” “To benefit other patients,” and “To support medical research” as significant motivations for sample donation after publicity, while fewer people chose the motivations “To reap financial rewards” and “To obtain social respect” after than before publicity. For the respondents without medical backgrounds, biobank and sample donation publicity significantly increased the percentage who chose “To help family, relatives, and future generations” and “To support medical research” as their main donation motivations (*P* < 0.001). However, 8.9% of respondents without medical backgrounds chose “To obtain social respect” before publicity, while the percentage decreased to 4.8% after publicity (*P* < 0.001). The details are shown in Table 4 and Figure 2.

**Table 4:** The main motivations and concerns of respondents about sample donation.

**Attitude**	**Respondents with medical**			**Respondents without medical**		
	**background (*****N*** = **464)**			**background (*****N*** = **481)**		
	**BP** ***N*** **(%)*^1^***	**AP** ***N*** **(%)*^1^***	***P^2^***	**BP** ***N*** **(%)*^1^***	**AP** ***N*** **(%)*^1^***	***P^3^***
**Main motivations of respondents about sample donation**						
To support medical research	84 (18.1)	111 (23.9)	< 0.001	75 (15.6)	102 (21.2)	< 0.001
To obtain social respect	69 (14.9)	39 (8.4)	< 0.001	43 (8.9)	23 (4.8)	< 0.001
To benefit other patients	95 (20.5)	130 (28.0)	< 0.001	131 (27.2)	130 (27.0)	1
To help family, relatives and future generations	113 (24.4)	155 (33.4)	< 0.001	141 (29.3)	155 (32.2)	< 0.001
To reap financial rewards	43 (9.3)	7 (1.5)	< 0.001	19 (4.0)	18 (3.7)	1
No willingness to donate	60 (12.9)	22 (4.7)	< 0.001	72 (15.0)	52 (10.8)	< 0.001
**Main concerns of respondents about sample donation**						
Leakage of personal information or biospecimens	130 (28.0)	185 (39.9)	< 0.001	221 (45.9)	224 (46.6)	0.25
Stigmatization	109 (23.5)	80 (17.2)	< 0.001	56 (11.6)	71 (14.8)	< 0.001
E-mail or telephone harassment after donation	118 (25.4)	85 (18.3)	< 0.001	111 (23.1)	113 (23.5)	0.5
Negative for health	44 (9.5)	42 (9.1)	0.5	50 (10.4)	47 (9.8)	0.25
No worries	63 (13.6)	72 (15.5)	0.004	43 (8.9)	26 (5.4)	< 0.001

BP, Before publicity; AP, After publicity.

1 Data presented as number and frequency.

2 McNemar's test was used to compare the change in motivations and concerns of respondents with medical background toward sample donation before and after publicity within the same group.

3 McNemar's test was used to compare the change in motivations and concerns of respondents without medical background toward sample donation before and after publicity within the same group.

Fisher's exact test was applied for those cases in which the expected frequencies were <5.

### Respondents' concerns about sample donation

Respondents' concerns before and after publicity mainly focused on “The leakage of personal information or biosamples,” “E-mail or telephone harassment after donation,” and “Stigmatization.” Among those with medical backgrounds, the percentage of respondents concerned about the leakage of personal information or biosamples increased from 28% before publicity to 39.9% after publicity, while the corresponding percentage of those without medical backgrounds increased from 45.9 to 46.6%. Biobank and sample donation publicity significantly relieved the concerns regarding e-mail or telephone harassment after donation for respondents with medical backgrounds (25.4 vs. 18.3%, *P* < 0.001). However, for respondents without medical backgrounds, biobank-related publicity had no impact on their concerns about e-mail or telephone harassment (23.1 vs. 23.5%, *P* = 0.5). Before biobank-related publicity, 23.5% of respondents with medical backgrounds worried about stigmatization, while this percentage reduced to 17.2% after publicity (*P* < 0.001). Among those without medical backgrounds, 11.6% of participants worried about stigmatization, while the percentage increased to 14.8% after publicity (*P* < 0.001). Regardless of whether the respondents had medical backgrounds, biobank and sample donation publicity did not aggravate or relieve respondents' concerns about the negative effect of sample donation on health (*P* > 0.05). The details are shown in Table 4 and Figure 2.

## Discussion

In a 1996 paper investigating the role of oxidative DNA damage as an independent risk factor in cancer, Loft and Poulsen first used the word “biobank” to refer to the use of human biological material (38). In 2004, the Chinese Biobank Study [Kadoorie Study of Chronic Disease in China (KSCDC)] was commenced and has a duration of 15–20 years (39–41). Extensive data collection has been undertaken with questionnaires, physical measurements, and collection and storage of blood samples (36, 39). Biobanks can provide high-quality samples and related information for diseases research, to optimize prevention, diagnosis, treatment and monitoring (42, 43). A large and growing number of samples and related information also offer opportunities to tackle the big data problems and population studies (44–46). In addition, biobanks are available for biomarker identification and drug discovery, development as well as validation (47, 48). Thus, biobanks have played an increasingly significant role in the development of precision and translational medicine. Although several studies have suggested that people with high education and from urban locations have a higher level of awareness about biobanking than people with lower education and from rural areas (49–53), this was not observed in our study. We considered that a lack of publicity aimed at the participants caused their lack of familiarity with biobanks and sample donation, which further led to their unwillingness to donate specimens. This is not beneficial for the improvement of medicine or the advancement of patient treatment. Studies have shown that one of the factors hampering willingness to donate is a lack of knowledge of biobanks and sample donation (40, 54, 55). This is consistent with our result shown in Table 1: the percentage of respondents who were unwilling to donate samples was nearly identical to that of respondents who were unfamiliar with biobanks and sample donation. Therefore, a range of effective publicity methods are needed to improve the awareness of biobanking, further promoting sample donation and medical development.

The internet is playing an increasingly significant role in public information. A survey conducted in four hospitals in Aleppo, Syria found the internet to be one of the most common sources of organ donation information (56). Another study on willingness for postmortem cornea donation by professionals in ophthalmology found that 53.9% of participants suggested the internet as a favorite source of information (57). The questionnaire in the present study was mainly distributed *via* the internet, which has the merits of low cost, large scale, high promotion, and high public acceptance (41, 58, 59). The questionnaire consisted of two parts, the questions and the sample donation publicity material, and aimed to improve participants' awareness of biobanks and sample donation as well as evaluate their attitudes toward sample donation before and after publicity provided with the questionnaires. Here, we discuss the questionnaire results in terms of: (1) the effectiveness of text-based, picture-based and lecture-based publicity in raising participants' willingness to donate; (2) factors that influence willingness to donate; and (3) respondents' motivations and concerns about donating biospecimens. Our results can provide people who want to collect human-derived samples for scientific research with suggestions of how to make a targeted explanation to relieve donators' worries caused by a lack of awareness for sample donation.

We distributed the questionnaires with the publicity materials of text and pictures to the interviewees. Respondents who attended a lecture related to biobanks and sample donation also received the questionnaires. Our results revealed that willingness to donate increased after each type of publicity. Another study also confirmed the effect of educational material about biobanks in improving participants' donation willingness (25). The present results suggest that the publicity material was easy to understand and appropriate for popularization of sample donation. We considered that a lack of publicity or improper publicity led to situation in which knowledge of sample donation is poor and rate of biobank participation is low, limiting specimen donation and hampering scientific and medical research based on human samples (60–63). Since effective publicity is of great significance in improving willingness to donate, the three methods were further compared to determine the most effective. Our results showed that picture-based publicity alone had a limited effect on sample donation willingness. Text-based publicity alone had a better effect but lecture-based publicity was the most effective in terms of increased donation rate. Lectures combine text, pictures and oral explanations to stimulate simultaneous visual, auditory, and advanced cognitive thinking. Moreover, the lecture explained the topic or terms more than once during the process, constantly strengthening participants' awareness of sample donation. In addition, the lecture created an opportunity for the participants and lecturer to subsequently discuss the topics, which helped to deepen participants' biobank-related knowledge. These features led to the superior effectiveness of the lecture compared to the other two methods. Although the lecture was better at prompting sample donation, it had limitations. Compared with the text material and picture material publicity methods, which could easily be distributed *via* the internet, the lecture required a venue, equipment, and a number of organizers, which made it complicated and inconvenient to conduct.

We also explored the factors affecting the effectiveness of text-based, picture-based, and lecture-based publicity in terms of improvement in willingness to donate. Our results revealed that participants with medical backgrounds were more willing to donate biospecimens after publicity than those without medical backgrounds. A previous study found higher willingness to donate a kidney among health science students than the general population (64). Since biobanks and sample donation are related to medical and scientific research, with which the general public are unfamiliar, people who with medical backgrounds may more easily understand sample donation publicity material. Therefore, the impact of biobank and sample donation publicity may be relatively high in recipients with medical backgrounds.

The main motivations affecting willingness to donate were “To help family, relatives and future generations,” “To benefit other patients,” and “To support medical research” in agreement with previous studies (61, 62, 65). The percentages of people with medical backgrounds motivated to donate samples “To reap financial rewards” and “To obtain social respect” decreased after publicity, with more respondents stating that they would donate samples to help others and promote medical research. The percentages of people without medical backgrounds motivated to donate samples “To obtain social respect” decreased after publicity, and more of those participants were willing to donate samples for scientific research. The results suggested that healthy young people, which accounted for the majority of our respondents, were positive about being altruistic and gaining a sense of responsibility to society (63). In addition, our publicity significantly improved willingness to donate and led some participants' motivations for donating samples to transform from self-serving to other-serving.

Our research also revealed factors hindering participation. In a previous study, some participants were concerned about privacy when they participated in scientific research (66), and the collection of biosamples from healthy people in China was challenging (52). We found that the leakage and loss of personal information was the main reason preventing people with and without medical backgrounds from participating. We think this result may be due to the severe information leakage that has been a common phenomenon in the internet age (67). Information inequalities and trust crises between biobanks, biobank staff, and donors have been major obstacles to biosample collection (52, 68–70). Moreover, we believe that this type of obstacle can be eliminated through biobank knowledge popularization and detailed pre-donation information exchange. Biosample collectors and publicists must correctly explain the security of informed consent, privacy protection, information protection and biosample use.

Our study compared the effectiveness of three publicity methods in prompting sample donation and discussed participants' motivations and concerns about donating specimens. However, it was not without limitations. First, our survey focused on young people ranging from 18 to 35 years old, rather than people of all ages. Given that two-thirds of our questionnaires were distributed through the internet and that older people may not be familiar with its operation, this may have had a negative effect on our data collection and analysis. Therefore, the data we collected were all from young participants, and despite the comparatively high participation rate, the results we obtained were not comprehensive and may not be generalizable to people of all ages. Second, although internet education has the merits of cost, large scale, high promotion, and high public acceptance, we cannot know whether the respondents who completed the questionnaires with carefully reading the content.

## Conclusion

As far as we know this is the first study to explore differences in effectiveness of publicity methods in raising willingness to donate biosamples in China. We found that biobank-related publicity based on text material, picture material, and a lecture all improved respondents' willingness and reduced concerns regarding sample donation to some extent, and lecture was the most effective. Our study provides suggestions for strategies to popularize biobanks and sample donation. In addition, our research reveals motivations and concerns about these topics, and these findings may help to improve sample donation and biobank systems, and thus support medical research.

## Data availability statement

The original contributions presented in the study are included in the article/Supplementary material, further inquiries can be directed to the corresponding author.

## Ethics statement

The studies involving human participants were reviewed and approved by the Center for Medical Ethics Committees for Protection of Human Subjects of the Third Xiangya Hospital of Central South University (No. 2016-S147). The patients/participants provided their written informed consent to participate in this study.

## Author contributions

The conception and design of the study were primarily conducted by ZZ and ZG. The drafting of the paper was mainly the responsibility of ZZ. FY, YH, and ZZ contributed to the material preparation and data collection and analysis. ZG and YH contributed to the distribution of the questionnaires. All authors have reviewed the analysis and interpretation of the data and contributed to the drafting of the manuscript, revising the manuscript, approved the final version to be published, and agree to be accountable for all aspects of the work. All authors have read and approved the final manuscript.

## Funding

The work was supported by the Research and Development Project of Central South University, China (Project No. 2018dcyj060), the Key Research and Development Programme of Hunan Province (No. 2018SK2090), the National Key Research and Development Programme of China (No. 2016YFC1201800), and Natural Science Foundation of Hunan Province, China (2022JJ40676).

## Conflict of interest

The authors declare that the research was conducted in the absence of any commercial or financial relationships that could be construed as a potential conflict of interest.

## Publisher's note

All claims expressed in this article are solely those of the authors and do not necessarily represent those of their affiliated organizations, or those of the publisher, the editors and the reviewers. Any product that may be evaluated in this article, or claim that may be made by its manufacturer, is not guaranteed or endorsed by the publisher.
