# Supplementary material for: Public awareness and attitudes toward biobank and sample donation: A regional Chinese survey
Source: Front Public Health. 2022 Nov 23;10:1025775. doi: 10.3389/fpubh.2022.1025775 (PMC9727410; doi:10.3389/fpubh.2022.1025775)
Supplement: Supplementary file 1 [file Data_Sheet_1.zip › Supplementary Figure S1, Text S1/Supplementary Figure S1.pdf]

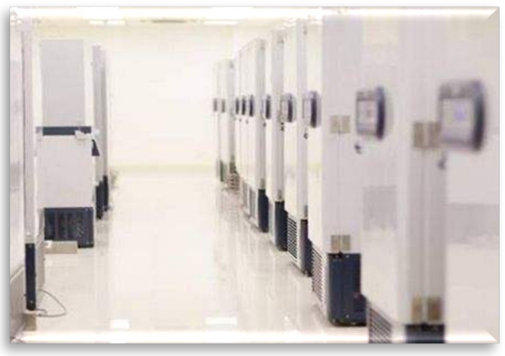
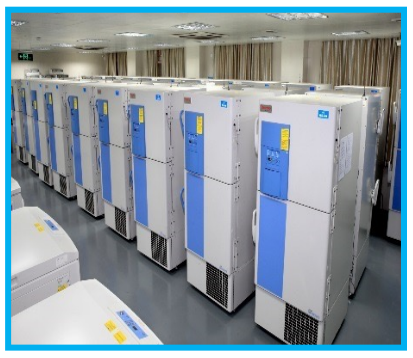
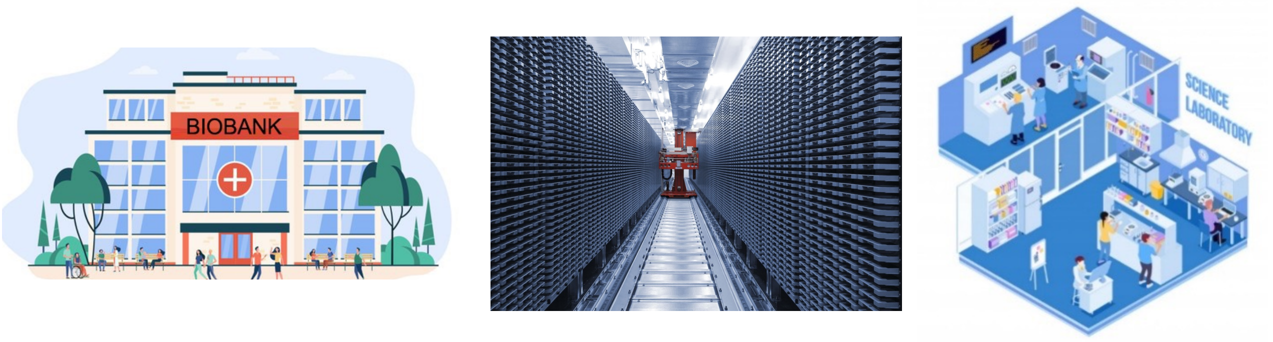

Biobank

Biobank is a kind of biological application system which persevered human biological material would be using for disease treatment and life science research.

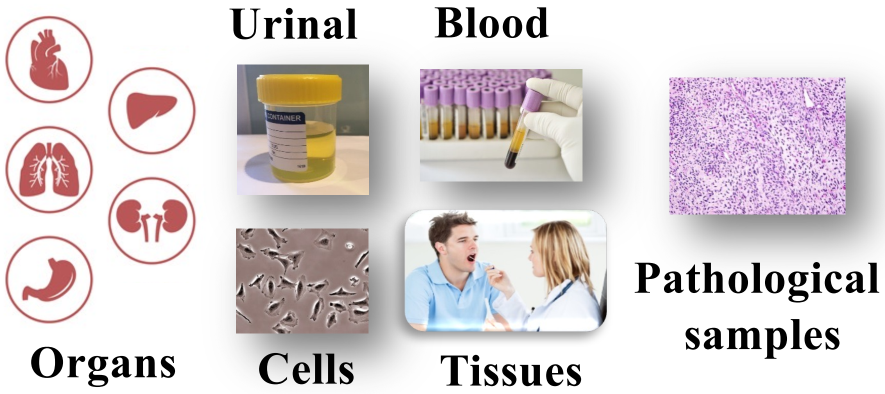

Donated Biological Samples

Human organs and tissues, blood, plasma, serum, body fluids or biological samples and the relative information.

NO pain or uncomfortable

Less pain of experimental animals

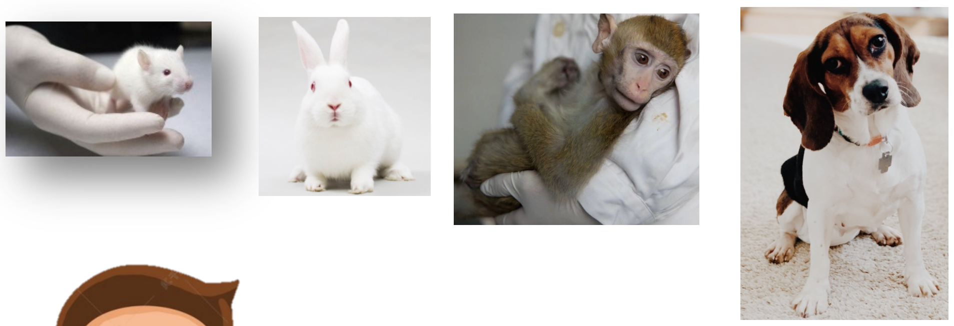

The Process of Donation

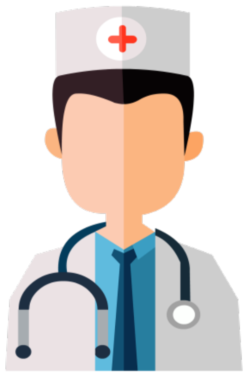

Communication

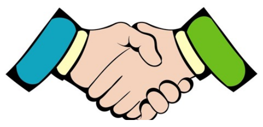
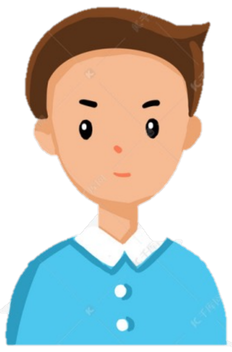

Sign the informed consent form

Donor's privacy will be protected to the extent required by law. The donated samples and information will be anonymized.

These biosamples and data will help medical diagnose, prevent and treat a range of diseases, such as diabetes, dementia, cancer, cardiovascular disease and infectious diseases.

Collect biosamples

Confirm the storage and usage

Any RISK？ NO

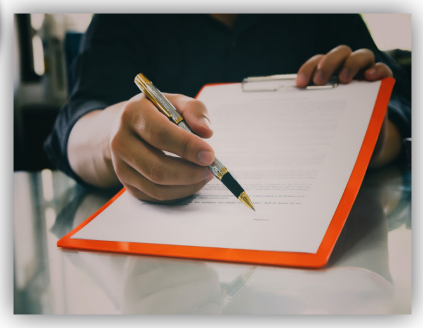

Sign informed consent form

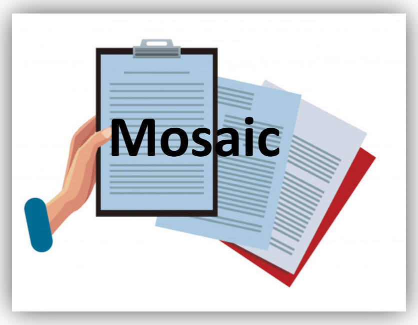

ID and information anonymous

The Usage of Biosamples

Genome mapping

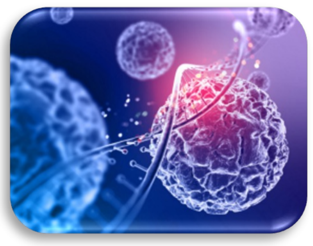

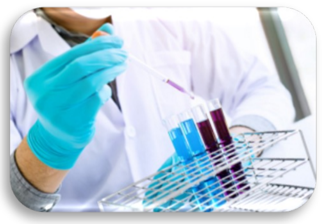

Drug development

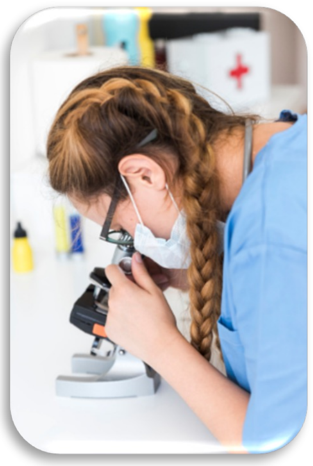

Medical diagnose

medical research

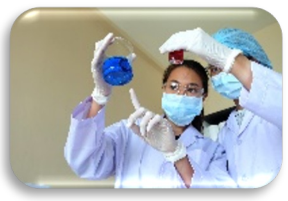

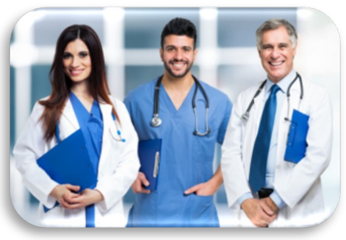

Medical education

Biobank

Medical usage

If the donor regret？

Withdraw consent at any time

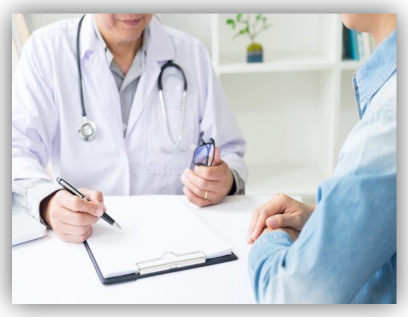

Withdraw consent at any time without giving a reason and sign a withdrawal statement.
The remaining samples and information will be destroyed and not use immediately at your request.

Learn More?
Scan the code below

code
