# Supplementary material for: Public awareness and attitudes toward biobank and sample donation: A regional Chinese survey
Source: Front Public Health. 2022 Nov 23;10:1025775. doi: 10.3389/fpubh.2022.1025775 (PMC9727410; doi:10.3389/fpubh.2022.1025775)
Supplement: Supplementary file 1 [file Data_Sheet_1.zip › Supplementary Figure S1, Text S1/Supplementary Text S1.pdf]

Text-based promotional materials：

1. What is a biobank?
Biospecimen bank, also known as biobank, mainly refers to the standardized collection, processing, storage and application of samples of biological macromolecules, cells, tissues and organs of healthy and diseased organisms (including human organs and tissues, blood, plasma, serum, body fluids or biological samples (DNA, RNA, proteins, etc.) and the clinical, pathological, treatment, follow-up, informed consent and other information related to these biosamples and the quality control, information management, and application systems.
In short, Biobank is a kind of biological application system which persevered human biological material would be using for disease treatment and life science research.

2. What are the categories of biobanks?
Commonly, there are tissue banks, organ banks, such as blood bank, cornea bank, bone marrow bank. It also has some cell biobanks unknown to the public, such as normal cells, genetic mutation cells, tumor cells and hybridoma cell strains (lines) of cell strains (lines) bank. In recent years, there are various stem cell banks such as umbilical cord blood stem cell banks, embryonic stem cell banks. In addition, there are also Genome banks for various human races and diseases. These biosample banks have played a very important role in promoting research on major diseases such as blood diseases, immune system diseases, diabetes, and malignant tumors.

3. As a health person, what is the relevance of donating biological samples to me?
Biosamples (such as blood, urine, stool, sputum, pleural fluid, cells, surgically removed tissues, etc.) generated during consultation and treatment may be used for medical research or education, drug development, disease treatment, etc. Especially for drug development and human diseases treatment, the biosamples contribute a lot. The increase of these biosamples and relative data will help diagnose, prevent and treat a range of diseases, such as diabetes, dementia, cancer, cardiovascular disease and infectious diseases. It will help to improve the prevention and treatment of diseases, and may help all people in the future, including you and your relatives and friends, and also promote medical development and improve people's healthy living standards.

4. Will there be any risk after I donate the biosample?
The process of donating samples will not cause any additional discomfort or health risks. Donated samples are collected along with samples collected for for disease diagnosis and treatment at the first time.

If you donate samples like blood, urine and stool, one more sample is encouraged to be preserved.
If you donate tissue samples, sample can only be collected under the conditions: (i) tissue samples are needed for disease diagnosis and treatment (ii) there are tissue sample left after using for pathological diagnosis.
You will not be re-traumatized during the donation process.
Donating samples will not affect your clinical treatment or increasing health risk.

5. Will my personal data or ID information be leaked?
The biobankers will protect your privacy to the extent required by law. Your donated samples and information will be anonymized, and sample users will not be able to access the identifiable personal data. Data from research using samples may be published publicly, but your name or identifiable information will not be published.

6. What if I regret it and don't want to donate anymore?
You can withdraw your consent at any time without giving a reason. If you want to withdraw your information and biosamples preserved in the biobank, you can contact the staff or the biobank. you will be required to sign a withdrawal statement at that time. Then, the preserved samples and information will be destroyed immediately and will not be used any more.

7. What's the process for me to donate samples now?
You need to sign an informed consent, which contains the above information about the biospecimen donation process and other information you need to be informed about. And the staff will confirm with you the preliminary matters related to the storage and usage of biosamples.
